# Biochar Improves Soil Aggregate Stability and Water Availability in a Mollisol after Three Years of Field Application

**DOI:** 10.1371/journal.pone.0154091

**Published:** 2016-05-18

**Authors:** Ningning Ma, Lili Zhang, Yulan Zhang, Lijie Yang, Chunxiao Yu, Guanghua Yin, Timothy A. Doane, Zhijie Wu, Ping Zhu, Xingzhu Ma

**Affiliations:** 1 Soil Ecology and Agro-ecological Engineering Research Center, Institute of Applied Ecology, Chinese Academy of Sciences, Shenyang 110164, People`s Republic of China; 2 Department of Land, Air, and Water Resources, University of California Davis, Davis 95616, United State of America; 3 Institute of Agricultural Resources and Environment, Jilin Academy of Agricultural Sciences, Changchun 130033, People`s Republic of China; 4 Institute of Soil Fertilizer and Environmental Resources, Heilongjiang Academy of Agricultural Sciences, Harbin, 150086, People`s Republic of China; 5 Graduate School, University of Chinese Academy of Sciences, Beijing 100049, People`s Republic of China; Old Dominion Univ., UNITED STATES

## Abstract

A field experiment was carried out to evaluate the effect of organic amendments on soil organic carbon, total nitrogen, bulk density, aggregate stability, field capacity and plant available water in a representative Chinese Mollisol. Four treatments were as follows: no fertilization (CK), application of inorganic fertilizer (NPK), combined application of inorganic fertilizer with maize straw (NPK+S) and addition of biochar with inorganic fertilizer (NPK+B). Our results showed that after three consecutive years of application, the values of soil bulk density were significantly lower in both organic amendment-treated plots than in unamended (CK and NPK) plots. Compared with NPK, NPK+B more effectively increased the contents of soil organic carbon, improved the relative proportion of soil macro-aggregates and mean weight diameter, and enhanced field capacity as well as plant available water. Organic amendments had no obvious effect on soil C/N ratio or wilting coefficient. The results of linear regression indicated that the improvement in soil water retention could be attributed to the increases in soil organic carbon and aggregate stability.

## Introduction

Mollisols have the notable characteristic of high native fertility, hence, even though they only occupy about 7% of the world’s total soil area, they nevertheless account for a much higher percentage of total crop production [1]. One of the world’s greatest expanses of Mollisols is located in northeast China, and supports a major sector of grain production in China. Continuous and intensive cultivation and suboptimal agricultural practices have led to serious deterioration of soil quality. According to survey data [2], after 40 years of continuous cultivation, the content of soil organic matter and total nitrogen in the top 30 cm of a typical Mollisol decreased by 49.7% and 61.2% respectively; field capacity decreased from 57.7% to 41.9%, while bulk density increased from 0.79 g cm^-3^ to 1.06 g cm^-3^.

Numerous studies have reported that organic amendments can increase soil fertility, ameliorate soil physical structure, enhance crop yields and improve soil resistance against erosion [3–5]. Among organic amendments, biochar has unique properties such as high porosity, low density, high carbon content, and the ability to retain additional carbon [6–8]. Many researchers have reached the consensus that biochar has the potential to improve soil properties and functions relevant to agronomic and environmental performance [9–15]. It is generally agreed that the mechanism of yield increase is the improvement in nutrient and water retention following application of biochar [16–18]. On the other hand, there are also studies that do not corroborate these findings. Major [19] found that addition of biochar produced from wood had no significant effect on either the water holding capacity or the saturated hydraulic conductivity of a clay soil. Similarly, Jeffery reported that soil water retention, aggregate stability, and field saturated hydraulic conductivity were not significantly affected by biochar amendment in a sandy soil [20]. Partly because of these discrepancies, the potential mechanisms behind changes in soil water retention characteristics after biochar application as an organic amendment still need to be further researched.

Studies on the effects of biochar application combined with fertilizer on nutrient and water retention in Mollisols and the associated mechanisms can contribute to improve soil fertility and sustainability of agroecosystems. We hypothesized that: (1) as an organic amendment, biochar has a positive influence on soil physical properties as well as nutrient and water retention; (2) these positive effects on nutrient and water retention are associated with increased soil aggregate stability.

## Materials and Methods

### Site description

The field experiment was conducted at the Black Soil Ecological Environment Key Field Research Station (42°57′ N, 148°57′ E), which is a long-term experiment established in 1979 and located in Gongzhuling, Jilin Province, China. is the site is supervised by the Jilin Academy of Agricultural Sciences who approved the present field research. The region has a temperate continental monsoon climate with hot and rainy summers and cold and dry winters. Mean annual temperature is 5.6°C and average annual precipitation is 595 mm. The soil at this site is classified as a Mollisol/Typic Hapludoll (USDA Soil Taxonomy) with 15.9 g kg^-1^ total organic carbon, 1.39 g kg^-1^ total nitrogen, and a clay loam texture.

The fields have been intensively cultivated for at least 50 years when the long-term experiment was initiated, and prior to the present study, the experimental fields were used for a soil nutrient depletion experiment, which was continuously cultivated with corn with no fertilization for 10 years.

### Experimental design

The three-year experiment (2012–2014) consisted of the following four treatments: no-fertilization (control, CK), application of inorganic fertilizer (NPK), combined application of inorganic fertilizer with maize straw (NPK+S), and combined application of inorganic fertilizer with biochar (NPK+B), arranged in a randomized complete block design with four replications. The main purpose was to compare the effects of fertilizer and fertilizer + organic amendments on fertility restoration in typical Mollisols. The study was set up in the field research station as a total of 16 micro-plots each with an area of 1.96 m^2^ (1.4 m× 1.4 m). Each set of four micro-plots was arranged in a row and treated as a replication, and the interval between neighboring replications was 1 m. In order to minimize surface runoff and horizontal diffusion of water and fertilizers, soils in each micro-plot was separated from surrounding soil by a 10-cm wide ‘brick wall’ with a 5-mm cement layer on each side from 50 cm below ground to 10 cm above ground. Fertilizer application rates were 135 kg N ha^-1^ (applied as urea), 67.5 kg P_2_O_5_ ha^-1^ (applied as triple superphosphate), and 67.5 kg K_2_O ha^-1^ (applied as potassium sulfate) for all the treatments except the control. Maize straw (containing 45% C) was incorporated into the topsoil at a rate equivalent to 11,100 kg ha^-1^ for the NPK+S treatment, representing complete return to the field of all the maize straw generated under conventional management. Maize straw was removed from the field in the other treatments. To make the C input in the NPK+B treatment equal to that of the NPK+S treatment, 7,800 kg ha^-1^ of commercial biochar (produced from maize straw and peanut hulls) with 64% C content were added in the NPK+B treatment. All P and K fertilizers, maize straw, biochar and 60% of the total N fertilizer were applied a week before sowing, and the remaining 40% of the N fertilizer was applied in early July (60 days after planting). Two or three maize seeds (Xianyu 335) were hole-sown in the first week of May with a plant spacing of 25 cm and row spacing of 65 cm in each plot. At the three-leaf stage of maize the young plants were thinned out and one plant in each hole was left to make the plant density in the micro-plots equivalent to 61,500 plants ha^-1^. Harvest of maize was around October 1st throughout the study period.

### Soil sampling

Soil samples were collected on Sep 29th 2014. Five randomized soil cores were taken in each micro-plot from a depth of 0-15cm with a soil auger, composited as one soil sample in the field, and stored in individual plastic bags. Meanwhile, an undisturbed soil block measuring about 2000 cm^3^ was taken from the same soil layer in each micro-plot and kept separately in a plastic box for soil aggregate and moisture characteristic analyses. All the soil samples were kept at 4°C until further analyses.

### Laboratory measurement

#### Soil physicochemical properties

Soil bulk density was determined from a core sample which was taken by driving a 100-cm^3^ stainless steel cylinder into the 5–10 cm soil layer. Two soil core samples were taken in each micro-plot for determination of soil bulk density. Soil organic carbon (SOC) and total nitrogen (TN) were determined by an Elemental Analyzer (Elementar, Vario EL).

#### Aggregate separation and mean weight diameter calculation

Undisturbed field-moist soil samples were air dried at room temperature until the soil moisture content reached about 8%, and then sieved through a 5 mm screen by gently pressing the clod of soil by hand. A total of 50 g processed soil were separated by a Retsch AS200 Control (Retsch Technology, Düsseldorf, Germany) with a nest of sieves of 2000 μm, 1000 μm and 250 μm. The sieves were mechanically shaken (amplitude 1.5 mm) for 2 minutes to obtain soil aggregates of different size fractions as follow: large macroaggregates (>2mm), macroaggregates (2-1mm), small macroaggregates (1–0.25mm) and microaggregates (<0.25mm) [21–22]. All of the isolated fractions were weighed, and were used to calculate the mean weight diameter (MWD) by the following equation:

$$\text{MWD}=\sum_{\text{i}}*{\text{P}}_{\text{i}}{\text{S}}_{\text{i}}$$

where P*i* is the proportion of the whole soil in the *i*th fraction and S*i* is the average diameter (mm) for particles in the *i*th fraction [23].

#### Soil water retention

Soil water retention was determined from undisturbed soil cores by the pressure membrane meter method (Soil moisture Equipment Co., USA). Soil cores and ceramic plates were placed into water and slowly saturated for 48h, then placed into pressure cells at tensions of 10, 20, 33, 100, 200, 400, 800, 1200 and 1500 kPa for 96 h to draw the soil-water characteristic curve (data not shown) The difference between volumetric moisture content at field capacity (-33 kPa) [24] and permanent wilting point (-1500 kPa) was taken as plant available water [25].

### Statistical analysis

Statistical analyses were performed by SPSS 19.1 software (SPSS Inc., Chicago, IL, USA). The effect of different treatments on soil MWD, plant available water, soil organic carbon, and total nitrogen was measured by one-way ANOVA analysis. Least significant difference (LSD) and Duncan tests were performed to assess differences between means. The relationship between SOC, MWD and AW were analyzed by Pearson correlation, Student’s t test was performed to assess differences between means. Differences at *P*<0.05 level were considered to be statistically significant.

## Results

### Soil physicochemical properties

After three years of fertilizer and organic amendment application, soil physicochemical properties changed significantly (Table 1). Both NPK+S and NPK+B treatments significantly reduced soil bulk density compared to CK and NPK. NPK decreased SOC significantly compared with the other three treatments. While NPK+B showed highest SOC and TN when compared with treatments without biochar application. Combined application of chemical fertilizer and biochar increased total nitrogen compared with NPK+S. There were no obvious differences in soil C:N ratio between different treatments.

**Table 1:** Soil physicochemical properties under different treatments.

	BD (g cm^-3^)	SOC (g kg^-1^)	TN (g kg^-1^)	C/N ratio
**CK**	1.36 ± 0.03a	18.69 ± 0.79a	1.54 ± 0.05c	11.70 ± 0.55a
**NPK**	1.32 ± 0.04a	17.51 ± 0.54b	1.63 ± 0.02ab	11.10 ± 0.20a
**NPK + S**	1.26 ± 0.03b	18.60 ± 0.41a	1.60 ± 0.06b	11.30 ± 0.58a
**NPK + B**	1.24 ± 0.04b	19.11 ± 0.86a	1.67 ± 0.03a	11.80 ± 0.47a

BD indicates bulk density; SOC and TN indicate soil organic carbon and total nitrogen respectively. Data are shown as means ± S.D.; Different letters indicate significant differences between fertilizer treatments (n = 4, *P* <0.05).

### Soil aggregate stability and mean weight diameter

Soil aggregate size distribution and mean weight diameter (MWD) in relation to inorganic fertilizer and organic amendment are shown in Table 2. Compared with CK, both the NPK+S and NPK+B treatments significantly increased the relative proportion of macro-aggregates (>2 mm) and the mean weight diameter, and reduced the relative proportion of micro-aggregates (<0.25 mm). The highest proportion of macro-aggregates was recorded in the NPK+B treatment; the proportion of >2 mm aggregates was 15%, 199%, and 35% higher than in the NPK+S, NPK, and CK treatments, respectively. The MWD of the NPK treatment was significantly lower than the other treatments. The proportion of the aggregates with a size of less than 1 mm was above 56% in the NPK treatment. Compared with the NPK treatment, both the NPK+S and NPK+B treatments significantly improved the stability of soil aggregates; however, biochar was more effective than maize straw.

**Table 2:** Aggregate size fractions and mean weight diameter (MWD) under different treatments.

Treatment	Aggregate size class (%)				MWD (mm)
	> 2 mm	2–1 mm	1–0.25 mm	< 0.25 mm	
**CK**	21.78±1.48c	34.64±3.55a	43.06±2.55b	0.52±0.11b	1.55±0.03c
**NPK**	14.77±1.70d	28.85±2.70b	55.29±4.06a	1.09±0.18a	1.30±0.07d
**NPK + S**	25.61±2.37b	33.98±2.28a	39.78±2.45bc	0.62±0.11b	1.66±0.06b
**NPK + B**	29.44±2.83a	32.35±2.41ab	37.68±1.14c	0.53±0.06b	1.75±0.06a

Data are shown as means ± S.D.; Different letters indicate significant differences between fertilizer treatments (n = 4, *P* <0.05).

### Soil water retention

The values of field capacity, wilting coefficient and plant available water under different treatments are given in Table 3. Different fertilization treatments had no effect on soil wilting coefficient. The NPK+B treatment significantly increased field capacity and plant available water when compared to the CK and NPK treatment. There was no significant difference between NPK and CK in soil field capacity, but the plant available water in the NPK treatment was significantly lower than that of CK (*P*<0.05). The values of soil field capacity and plant available water followed the order: NPK<CK<NPK+S<NPK+B.

**Table 3:** Soil water retention under different treatments.

	Field Capacity (cm^3^ cm^-3^)	Wilting Coefficient (cm^3^ cm^-3^)	Plant Available Water (cm^3^ cm^-3^)
**CK**	41.35 ± 1.32b	28.99 ± 1.23a	12.36 ± 1.05b
**NPK**	41.25 ± 1.43b	30.69 ± 1.87a	10.57 ± 0.71c
**NPK + S**	41.88 ± 0.74ab	28.65 ± 0.58a	13.23 ± 0.28ab
**NPK + B**	43.78 ± 1.82a	30.09 ± 1.52a	13.70 ± 0.86a

Data are shown as means ± S.D.; Different letters indicate significant differences between treatments (n = 4, *P* <0.05).

## Discussion

A number of studies have reported positive effects of biochar on improving soil fertility and increasing crop yield [9,10,26,27]. Chan (2007) found that even at a high application rate (100 t ha^-1^), biochar amendment alone did not increase radish dry matter yield [26]. However, an increase of 95% in radish yield was observed when N fertilizer was applied in combination with biochar. Similarly, Jones (2012) reported an increase in yield when N fertilizer and biochar (100 t ha^-1^) were applied together [28]. These studies reflect the potential of biochar to increase the nitrogen fertilizer use efficiency of plants. In our study, applications of biochar and fertilizer contributed to a significant increase in soil organic carbon, but otherwise exhibited no substantial effects on soil total nitrogen and C:N ratio compared with the NPK treatment. This might be due to the lower application rate of biochar (7.8 t ha^-1^) in comparison with the aforementioned studies. Liu (2014) investigated the effects of different application rates of biochar (0, 2.5, 5, 10, 20, 30 and 40 t ha^-1^) on rapeseed and sweet potato yields, aggregate stability, soil organic carbon, total N and C:N ratio. Their study demonstrated that significant increments in soil organic carbon and total N were only possible at the highest biochar application rate (40 t ha^-1^) [29]. In our study, although combined application of biochar plus NPK did not increase soil total N compared with that of NPK treatment, it did cause a more notable increase in soil organic carbon and decrease in soil bulk density; this role may be of greater value for fertility restoration of Mollisols.

**Fig 1:**
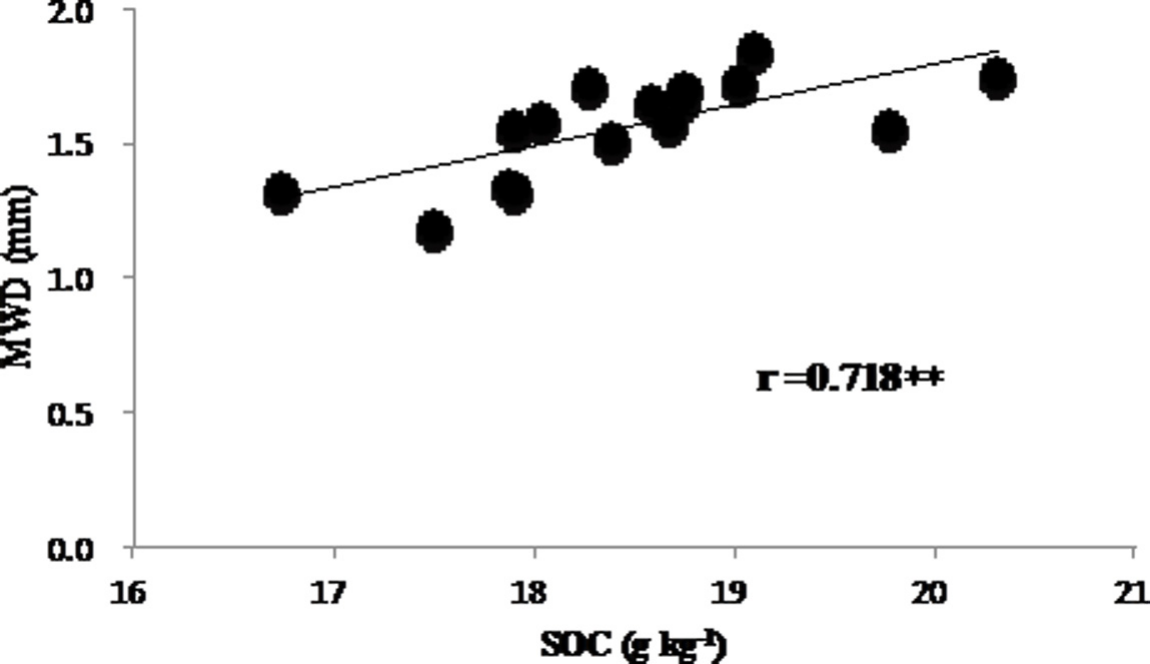
Linear regression between soil organic carbon (SOC) and soil mean weight diameter (MWD). “r” indicates pearson correlation coefficient, and ** indicate significance at *P*<0.01.

There are contradictory conclusions regarding the impacts of biochar on soil aggregates. Liu (2012) conducted an incubation experiment to detect the effects of biochar on soil aggregates in four types of soils typical of the Loess Plateau in China [30]. They found that biochar application could increase the water-stable aggregate content of silt loam soils, but had no significant influence on aggregate formation and stability of sandy loam soils. Hardie (2014) reported that the application of 47 megagram ha^-1^ of biochar made from the green waste of whole acacia trees had no significant effect on the stability of the 1–2 mm aggregates in a planosol [31]. In our study, the most dramatic effect of biochar was its role in increasing the proportion of macro-aggregates and therefore the mean weight diameter (MWD). Biochar influences soil aggregation due to interactions with soil organic matter, minerals and microorganisms [32]. High CEC made biochar more effective as a binding agent for organic matter and minerals to form macro-aggregates [32]. A significantly positive correlation between soil organic carbon and mean weight diameter of aggregates (Fig 1) suggests that the increase in SOC may contribute to aggregate stability, which is also reported by several other publications [33–34].

**Fig 2:**
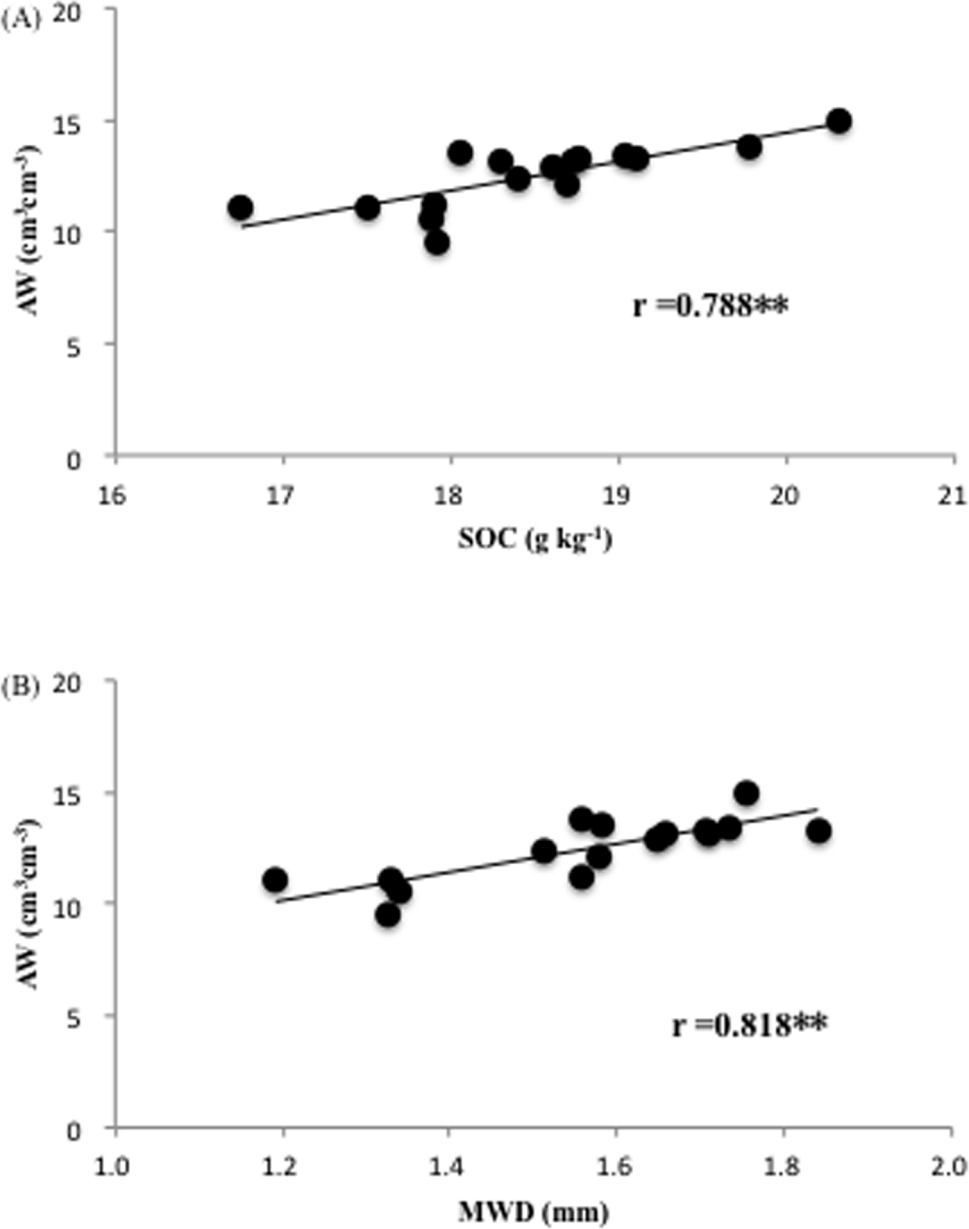
**(A) Linear regression between soil organic carbon (SOC) and available water (AW). (B) Linear regression between soil mean weight diameter (MWD) and available water (AW).** “r” indicates pearson correlation coefficient, and ** indicate significance at *P*<0.01.

The results of linear regression further revealed a significant positive correlation between plant available water and soil organic carbon, and between plant available water and mean weight diameter of aggregates (Fig 2). Verheijen (2010) suggested that water retention of soil is determined by the distribution and connectivity of pores in the soil medium, which is largely regulated by soil particle size (texture), combined with structural characteristics (aggregation) and SOM content [32]. The positive effect of biochar on soil physical properties and soil water retention was well documented. Abel (2013) studied the effects of biochar and hydrochar on physical properties and water retention characteristics of a sandy soil, and showed that application of biochar decreased bulk density, but increased total pore volume, water content at the permanent wilting point, as well as available water capacity [35]. Masulili (2010) showed that organic amendments, especially biochar, significantly increased total soil porosity and plant available water [36]. The authors attributed this to the formation of soil aggregates, which is confirmed by the present study. There have been contrasting conclusions about the effect of biochar on soil water retention [19–20]. Therefore, the effect of biochar application on soil water retention needs to be assessed on a case-by-case basis. Attention should not only be paid to biochar characteristics (e.g. feedstock and pyrolysis conditions), but also to soil type and application strategies [32].

## Conclusion

Our study demonstrates some of the beneficial effects of biochar as an organic amendment for sustainable agriculture of Mollisols in northeast China. Three-year consecutive combined application of biochar and inorganic fertilizer significantly improved soil physical properties: bulk density decreased, while the relative proportion of soil macro-aggregates and mean weight diameter of aggregates increased. Soil organic carbon, total nitrogen and plant available water in biochar-treated plots were obviously higher than those in the non-fertilized treatment, which indicate that biochar, in combination with fertilizer, can effectively improve the ability of soil to retain nutrients and water. The significant relationships between soil organic carbon and available water, as well as between mean weight diameter of aggregates and available water, confirm the close connection between improvement of soil structure and its ability to supply water. Biochar is therefore an ideal amendment for improving certain key properties of degraded Mollisols, as it can provide tangible benefits in only several years and at a relatively modest rate of application.

## Supporting Information

S1 Data The original data for generating Fig 1 in the manuscript. (XLSX)

S2 Data The original data for generating Fig 2 in the manuscript. (XLSX)

S3 Data The original data for generating Table 1 in the manuscript. (XLSX)

S4 Data The original data for generating Table 2 in the manuscript. (XLSX)

S5 Data The original data for generating Table 3 in the manuscript. (XLSX)
